# Retraction Note: Effects of DA-5513 on alcohol metabolism and alcoholic fatty liver in rats

**DOI:** 10.1186/s42826-020-00047-z

**Published:** 2020-06-11

**Authors:** Jae Young Yu, Hanh Thuy Nguyen, Chul Soon Yong, Hyoung Geun Park, Joon Ho Jun, Jong Oh. Kim

**Affiliations:** 1 Department of Formulation Development, Dong-A Pharmaceutical Co Ltd., Yongin, South Korea; 2 College of Pharmacy, Yeungnam University, Gyeongsan, South Korea; 3 National Institute of Pharmaceutical Technology, Hanoi University of Pharmacy, Hanoi, Vietnam

**Retraction Note: Lab Anim Res (2018) 34: 49–57**

**https://doi.org/10.5625/lar.2018.34.2.49**

This article [1] has been retracted at the request of authors. It has come to our attention that Seoul National University co-own the data presented in the current study with Dong-A Pharma. Unfortunately, we did not obtain permission to publish the data prior to publication and did not give proper credit to all collaborators on this research project. In addition, authors have been unable to replicate the results presented after conducting further experiments using DA-5513. We are therefore concerned about the reliability of the findings.

All authors agreed to this retraction.
